# Impact of time to start of tranexamic acid treatment on rebleed risk and outcome in aneurysmal subarachnoid hemorrhage

**DOI:** 10.1177/23969873241246591

**Published:** 2024-04-12

**Authors:** Menno R Germans, Maud A Tjerkstra, René Post, Amy Brenner, Mervyn DI Vergouwen, Gabriël JE Rinkel, Yvo BWEM Roos, René van den Berg, Bert A Coert, W Peter Vandertop, Dagmar Verbaan, for the ULTRA Investigators

**Affiliations:** 1 Department of Neurosurgery, Clinical Neuroscience Center, University Hospital Zurich, Zurich, Switzerland; 2 Department of Neurosurgery, Amsterdam University Medical Centers Location University of Amsterdam, Amsterdam, The Netherlands; 3 Amsterdam Neuroscience, Neurovascular Disorders, Amsterdam, The Netherlands; 4 Clinical Trials Unit, Department of Population Health, London School of Hygiene and Tropical Medicine, London, UK; 5 Department of Neurology and Neurosurgery, UMC Utrecht Brain Center, University Medical Center Utrecht, Utrecht University, Utrecht, The Netherlands; 6 Department of Neurology, Amsterdam University Medical Centers location University of Amsterdam, Amsterdam, The Netherlands; 7 Department of Radiology and Nuclear Medicine, Amsterdam University Medical Centers Location University of Amsterdam, Amsterdam, The Netherlands

**Keywords:** Stroke, subarachnoid hemorrhage, randomized controlled trial, antifibrinolytic agent, time interval

## Abstract

**Introduction:**

The ULTRA-trial investigated effectiveness of ultra-early administration of tranexamic acid (TXA) in subarachnoid hemorrhage (SAH) and showed that TXA reduces the risk of rebleeding without concurrent improvement in clinical outcome. Previous trials in bleeding conditions, distinct from SAH, have shown that time to start of antifibrinolytic treatment influences outcome. This post-hoc analysis of the ULTRA-trial investigates whether the interval between hemorrhage and start of TXA impacts the effect of TXA on rebleeding and functional outcome following aneurysmal SAH.

**Patients and methods:**

A post-hoc comparative analysis was conducted between aneurysmal SAH patients of the ULTRA-trial, receiving TXA and usual care to those receiving usual care only. We assessed confounders, hazard ratio (HR) of rebleeding and odds ratio (OR) of good outcome (modified Rankin Scale 0–3) at 6 months, and investigated the impact of time between hemorrhage and start of TXA on the treatment effect, stratified into time categories (0–3, 3–6 and >6 h).

**Results:**

Sixty-four of 394 patients (16.2%) in the TXA group experienced a rebleeding, compared to 83 of 413 patients (19.9%) with usual care only (HR 0.86, 95% confidence interval (CI): 0.62–1.19). Time to start of TXA modifies the effect of TXA on rebleeding rate (*p* < 0.001), with a clinically non-relevant reduction observed only when TXA was initiated after 6 h (absolute rate reduction 1.4%). Tranexamic acid treatment showed no effect on good outcome (OR 0.96, 95% CI: 0.72–1.27) with no evidence of effect modification on the time to start of TXA (*p* = 0.53).

**Discussion and conclusions:**

This study suggests that the effect of TXA on rebleeding is modified by time to treatment, providing a protective, albeit clinically non-relevant, effect only when started after 6 h. No difference in functional outcome was seen. Routine TXA treatment in the aneurysmal SAH population, even within a specified time frame, is not recommended to improve functional outcome.

## Graphical abstract

**Graphical abstract:**
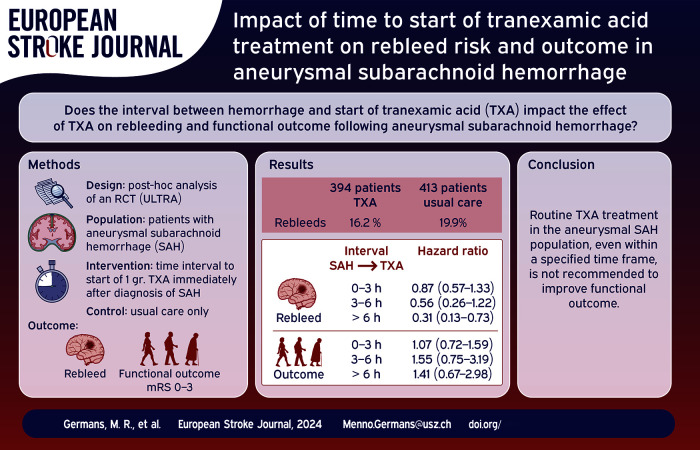

## Introduction

One factor with a major impact on poor outcome in aneurysmal subarachnoid hemorrhage (aSAH) is recurrent hemorrhage (“rebleeding”) of the aneurysm.^1,2^ Its incidence is approximately 12%–16% in the first 24 h after the initial hemorrhage with the highest rebleed rate in the first hours after the initial hemorrhage.^1–5^ The emergency medical management of aSAH has been extensively evaluated and procedures to shorten the time to diagnosis and treatment have been investigated.^6^ Nevertheless, procedures to shorten the time intervals have been difficult to achieve because of the delay in transport of patients to a hospital for confirmation of the diagnosis and treatment in only highly specialized centers. Moreover, because of the high rebleed rate in the first few hours, even expedited aneurysm treatment did not reach sufficient reduction of rebleeds to improve overall outcome.^7–9^

An alternative treatment strategy aimed at a reduction of rebleeds is administration of an antifibrinolytic agent.^10^ The evidence for an effect of an antifibrinolytic agent on reducing rebleeds in aSAH is strong (risk ratio 0.65, 95% confidence interval (CI): 0.47–0.91), however, no positive effect on functional outcome after at least 3 months after the hemorrhage was achieved.^11^ Studies in trauma and post-partum hemorrhage showed evidence of a survival benefit with administration of an antifibrinolytic agent within 3 h of bleeding onset, with a larger effect when administered earlier.^12^ With this in mind, it is possible that the delay to first administration of the antifibrinolytic agent in aSAH patients, and therefore less impact on the reduction of rebleeds, may be a factor attributing to the failure of improving overall outcome. To investigate this, we did a post-hoc analysis of the randomized controlled ULTRA-trial.^13^ The aim of this study is to investigate whether the time between initial aSAH and start of tranexamic acid (TXA) treatment influences the effect of TXA on rebleed rate and functional outcome at 6 months after the hemorrhage.

## Methods

This manuscript is presented following the CONSORT guidelines.

### Eligibility criteria

The data for this project are originating from the ULTRA-trial. This multicenter, randomized, open-label trial with blinded endpoint assessment was conducted in the Netherlands between July 2013 and July 2019 and enrolled 955 individuals with SAH.^13^ For a detailed description of methods and ethical approval of the original study, we refer to the published protocol and statistical analysis plan.^14,15^ The TXA group received 1 g of TXA intravenously immediately after the diagnosis of SAH by non-contrast CT, followed by a continuous infusion of 1 g every 8 h and stopped immediately before aneurysm treatment or 24 h after start of the agent, whichever came first. This agent was given in addition to usual care. The control group received only usual care according to recent guidelines.^16^

As patients with SAH due to an aneurysm are at risk for a rebleed, we only included the patients with a confirmed aneurysm in additional cerebrovascular imaging. In the ULTRA population 813 individuals suffered an aSAH, which is 85% of the total ULTRA population. Details of this subgroup have recently been published.^17^

### Baseline data

For a detailed description of the baseline characteristics, we refer to the publication of the primary results of the ULTRA-trial.^13^ Baseline characteristics for this project include demographic, admission, radiological and treatment information, together with time intervals. For this study, age was categorized into three groups according to known age distribution among aSAH patients: 20–49 years, 50–69 years, and ⩾70 years.

### Time intervals

Each time point between initial hemorrhage onset and aneurysm treatment was registered according to strict definitions described in Supplemental File 1. Time intervals were calculated by assessing the difference between the respective times. Because some times were missing, we decided not to add up each individual interval but to calculate the interval between the respective times. The parameter “at risk for rebleed” was defined as the interval between the (approximated) time of initial hemorrhage and the start of aneurysm treatment, in hours.

Previous studies in the Netherlands have shown that half of all rebleeds occur within 3 h and two thirds within 6 h after the initial hemorrhage.^3,5^ Literature regarding early administration of TXA in various indications showed that it may only be beneficial when given within 3 h after bleeding onset.^12,18,19^ In the ULTRA-trial, the majority of SAH was diagnosed within 3 h after the initial signs and symptoms of the hemorrhage (median 93 min, interquartile range (IQR): 65–165). Taking this into consideration we decided to stratify the interval between initial hemorrhage and start of TXA treatment into three categories: early (0–3 h), middle (3-6 h) and late (>6 h). Because the control group did not receive study medication, a surrogate time interval was calculated. Therefore, the median time between randomization and start of TXA treatment in the TXA group was added to the time of randomization in the control group to calculate the surrogate time interval for each patient.

### Outcomes

The primary outcome was a rebleed, defined as a sudden neurological deterioration with change in vital parameters suggestive for rebleed (possible rebleed) and presence of more SAH on CT than in a previous investigation (CT-proven rebleed). Also, possible rebleeds before the SAH diagnosis and during endovascular or surgical treatment were included.

The secondary outcome was functional outcome assessed by the modified Rankin Scale (mRS) at 6 months. In the ULTRA-trial, this outcome assessment was done through a validated telephone interview by trained research nurses who were masked to treatment allocation.^20^

### Statistical analysis

The functional outcome (mRS) was dichotomized into 0–3 (good outcome, ranging from no deficits to moderate disability) versus 4–6 (poor outcome, ranging from moderately severe disability to dead). Baseline data were presented as numbers and proportions (%), and group differences were compared using the χ^2^-test. As there was little missing data in the ULTRA-trial we decided to exclude cases with missing data. Continuous data were assessed for normality with the skewness and kurtosis tests for normality and normal distribution plot. An independent *t*-test and presentation of means with standard deviation (SD) were done for a parametric distribution, whereas in a non-parametric distribution the Mann-Whitney *U* test and medians with interquartile ranges (IQR) were presented. The data are analyzed according to the as-treated analysis.

#### Univariable regression

Rate ratios (RR) and 95% CI were calculated comparing those who received TXA with those who received standard care (as-treated analysis). Rate of rebleeding was calculated as the number of rebleeds divided by patient-days at risk for rebleed and compared with the χ^2^-test. To assess potential confounding variables, we performed Mantel-Haenszel analyses. If crude and adjusted RR differed by around 10% or more, the stratifying variable was considered a confounder and included in the multivariable regression analysis. An identical confounder analysis was done for functional outcome, with odds ratios (OR) as the effect measure.

#### Multivariable regression

Kaplan-Meier failure curves and the log-rank test were used to compare event rates in patients who received TXA and those who did not.

A Cox regression model with hazard ratios (HR) and 95% CI was used to investigate the rate of rebleed between patients with and without TXA administration. In this model, confounding variables are added according to the confounder assessment mentioned above. The (estimated) time of initial hemorrhage is taken as entry, with rebleed as failure event and exit when aneurysm treatment is started. If the aneurysm was not treated, the time of death or a maximum time of 2 weeks of follow-up after randomization to TXA or standard care is used as exit. Finally, we conducted a subgroup analysis to explore the effect of TXA on rebleed rate in relation to the (estimated) time between initial hemorrhage and start of TXA treatment. Therefore, we compared the rebleed hazard ratio across three time categories: early (0–3 h), middle (3–6 h) and late (>6 h). We fitted an interaction term for time to start of TXA or surrogate time interval and compared model fit with the likelihood ratio test.

An identical analysis was done for functional outcome (mRS) with OR as effect measure.

#### Sensitivity analysis

Studies have shown that some rebleeds occur very early and these are strongly associated with poor outcome.^1,5^ Therefore, it is possible that rebleeds occurred before the confirmation of aSAH with CT, which could not have been prevented with immediate start of TXA treatment after the diagnosis. We decided to perform a sensitivity analysis on both outcomes with exclusion of rebleeds occurring before the diagnosis of aSAH by CT.

The statistical analyses were performed with STATA (StataCorp LLC, Texas, USA; version 16.1). Due to the post hoc data analysis and multiple comparisons, the Holm–Bonferroni method is applied for controlling the family-wise error rate.^21^ The significance levels of each analysis are adjusted with a type I error (α) of 5%.

### Standard protocol approvals, registrations, and patient consents

The protocol of the ULTRA-trial was approved by the Medical Ethics Committee of the Amsterdam University Medical Center, location AMC. For a detailed description of the ethical approval, we refer to the previously published articles.^13–15^ Written consent was obtained from all participants or their legal representatives as explained in previous publications.^14,15^ The ULTRA-trial was registered at ClinicalTrials.gov (2012-000343-26; registered on February 17, 2016) and the Netherlands Trial Register (NTR3272; registered on January 25, 2012).

### Data availability

Investigators may request access to anonymized individual patient data and redacted trial documents including raw datasets, analysis-ready datasets, trial protocols, annotated case report form, statistical analysis plan, dataset specifications, and clinical trial report 20 months after trial is complete. Prior to use of the data, proposals need to be approved by an independent review panel at www.clinicaltrialsett.com and a signed data sharing agreement will then be approved. All documents are for a predetermined time, typically 12 months.

## Results

### Baseline characteristics

In the 813 aSAH patients of the ULTRA-trial, 16 (3.9%) patients in the TXA group did not receive TXA, whereas two (0.5%) patients in the control group did receive TXA. In one patient, allocated to the TXA group, it was uncertain whether tranexamic acid was administered and this patient was therefore excluded from the analysis.^17^ In the current as-treated analysis, 394 (48.5%) patients received TXA treatment and 418 (51.5%) patients received usual care.

Six patients (of whom two received TXA) withdrew the informed consent and those data were excluded from the 6 months follow-up analysis (see Supplemental Figure S1). The baseline characteristics were equally divided between groups (Tables 1 and 2). Time intervals could not be calculated for 29 patients, largely because the randomization time was not saved in the randomization software due to a software error (n = 10). Patients with missing time intervals were also excluded from further analysis.

**Table 1.** Baseline characteristics of 812 patients with aneurysmal subarachnoid hemorrhage.

Variable	TXA and usual care *N* = 394	Usual care only *N* = 418	*p*-Value,^*^
Age (years), mean (SD)	58.4 (12.8)	58.4 (12.2)	0.96
Sex (female)	285 (72)	290 (70)	0.44
WFNS^†^			0.58
I	126 (33)	148 (36)	
II	77 (20)	75 (18)	
III	23 (6)	16 (4)	
IV	84 (22)	95 (23)	
V	77 (20)	81 (20)	
I–III	226 (58)	239 (58)	0.82
IV–V	161 (42)	176 (42)	
Fisher grade			0.21
II	21 (5)	15 (4)	
III	99 (25)	124 (30)	
IV	274 (70)	279 (67)	
Medication use			
Platelet inhibitor	48 (12)	49 (12)	0.55
Anticoagulation	13 (3)	14 (3)	0.57
Location of aneurysm^‡^			0.75
Anterior circulation	261 (68)	273 (67)	
Posterior circulation	124 (32)	136 (33)	
Treatment modality^§^			0.53
Endovascular	264 (67)	265 (63)	
Clipping	80 (20)	95 (23)	
None	49 (12)	58 (14)	
Time category (h)			0.62
0–3	194 (49)	211 (51)	
3–6	122 (31)	117 (28)	
>6	78 (20)	90 (22)	
Rebleeding per time category (h)			0.62
0–3	38 (10)	49 (12)	
3–6	17 (4)	21 (5)	
>6	8 (2)	12 (3)	
Treatment center			0.91
A	177 (45)	182 (44)	
B	73 (19)	87 (21)	
C	64 (16)	66 (16)	
D	25 (7)	26 (6)	
E	21 (6)	25 (6)	
F	17 (5)	18 (4)	
G	13 (3)	11 (3)	
H	2 (1)	3 (1)	
other	2 (1)	0	

SD: standard deviation; TXA: tranexamic acid; WFNS: World Federation of Neurosurgical Societies.

Data are presented as n (%), unless noted otherwise. Percentages may not total 100 because of rounding.

* χ^2^-test for all variables except age: independent *t*-test.

† WFNS score: missing in 10 (1.2%).

‡ Location of aneurysm: missing in 18 (2.2%).

§ Treatment modality: one individual with two potentially causative aneurysms, of which one was clipped and one was treated endovascularly.

**Table 2.** Time of events for 811 patients with aneurysmal subarachnoid hemorrhage.

Time interval	Combined *N* = 811	TXA and usual care *N* = 394	Usual care only *N* = 417	*p*-Value,^*^
Hemorrhage to CT	90 (63–164)	91 (63–168)	87 (63–159)	0.86
Hemorrhage to randomization	166 (111–297)	170 (111–284)	164 (111–308)	0.67
Hemorrhage to rebleed^†^	225 (109–478)	222 (90–410)	225 (120–585)	0.46
Hemorrhage to aneurysm treatment^‡^	996 (507–1470)	1001 (584–1485)	979 (433–1470)	0.41

CT: computed tomography; TXA: tranexamic acid.

Data presented as medians (interquartile ranges) and in minutes. Hemorrhage indicates the time of initial hemorrhage.

Missings: Hemorrhage to CT: 1 (0.1%); Hemorrhage to randomization: 9 (1.1%); Hemorrhage to rebleed: 1 (0.7%); Hemorrhage to treatment: 1 (0.1%).

* Mann-Whitney *U* test for all variables.

† 143 rebleeds occurred (62 in TXA group, 81 in control group).

‡ 698 aneurysm were treated (342 in TXA group, 355 in control group).

Times could be assessed in a total of 811 patients, with an exact or estimated time of initial hemorrhage in 731 (90%) patients, whereas in 80 (10%) patients it was estimated by calculations. See Supplemental Table S1 for an overview of the median time intervals to start of TXA treatment and its surrogate time interval in those patients who did not received TXA treatment.

### Analysis of rebleeds

A rebleed was registered in 64 of 394 (16.2%) patients who received TXA treatment and in 83 of 418 (19.9%) in those who received usual care, with confirmation by CT in 102 (69%) cases. The distribution of rebleeds among time categories was 87 (60%), 38 (26%), and 20 (14%), for 0–3, 3–6, and >6 h, respectively. The total time at risk for rebleed was comparable between both groups.

The unadjusted analysis showed a rebleed rate of 0.08 (95% CI: 0.07–0.11) per person-day in the TXA group and 0.11 (95% CI: 0.09–0.13) per person-day in the control group (RR 0.80, 95% CI: 0.58–1.11). See Supplemental Table S2 for an overview of the unadjusted rates and RR for rebleed by baseline variables. The data contained no confounders.

**Figure 1.**
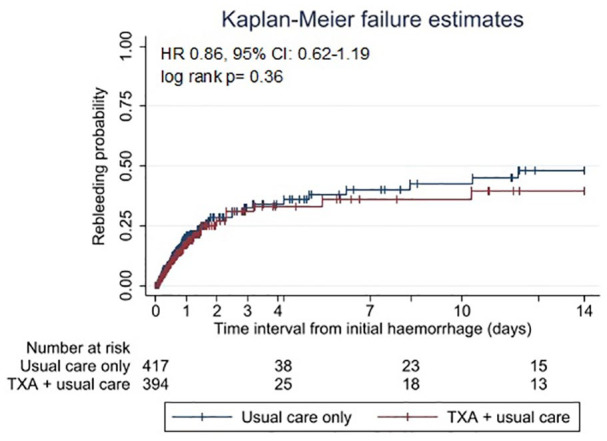
Kaplan-Meier failure graph for probability of rebleeds within 2 weeks after the initial hemorrhage in 811 aneurysmal subarachnoid hemorrhage patients. Vertical lines indicate censoring of cases. TXA: tranexamic acid.

**Figure 2.**
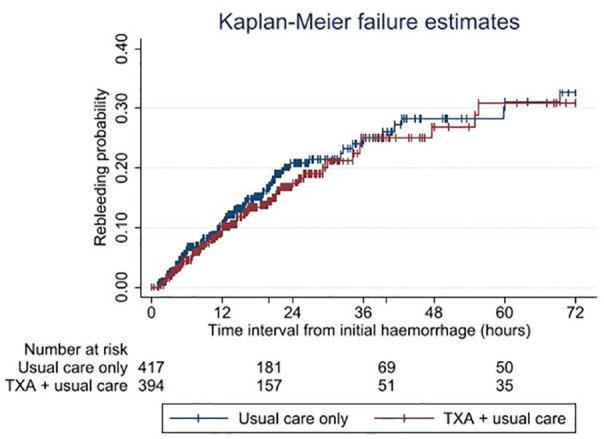
Kaplan-Meier failure graph for probability of rebleeds within 72 h after the initial hemorrhage in 811 aneurysmal subarachnoid hemorrhage patients. *Y*-axis is restricted between 0 and 0.40 to better illustrate the lines. Vertical lines indicate censoring of cases. TXA: tranexamic acid.

The Kaplan-Meier survival curve (Figures 1 and 2) showed comparable lines with no difference between the curves (log-rank test *p* = 0.36).

The Cox regression model showed no evidence for an overall reduction of rebleeds by TXA (hazard ratio [HR] 0.86, 95% CI: 0.62–1.19) (see Table 3). The subgroup analysis by time category showed a protective effect of TXA only when time to treatment was longer than 6 h after the initial hemorrhage (HR 0.31, 95% CI 0.13–0.73; absolute rate reduction 1.4%). There was evidence that the effect of TXA on rebleeding differed by time to treatment (likelihood ratio test: *p* < 0.001).

**Table 3.** Cox regression model with effect on rebleeding.

	Number of rebleeds (% of total rebleeds)	Hazard ratio (95% CI)	*p*-Value,^*^
Tranexamic acid	64 (43.8)	0.86 (0.62–1.19)	0.36
Time category (h)			
0–3	79 (58.5)	0.87 (0.57–1.33)	0.52
3–6	38 (28.1)	0.56 (0.26–1.22)	0.15
>6	18 (13.3)	0.31 (0.13–0.73)	0.01

CI: confidence interval; TXA: tranexamic acid.

* Wald-test.

After excluding rebleeding occurring before diagnosis, in an analysis on 775 patients, there was no evidence of a reduction in rebleeds with TXA (RR 0.80, 95% CI: 0.55–1.16). There was strong evidence for effect modification by time to treatment (*p* < 0.001). Otherwise, the results remained grossly the same (see Table S3).

### Functional outcome at 6 months

The modified Rankin Scale was available in 803 individuals. A good functional outcome was seen in 470 (58%) individuals and case fatality was 28% (224 of 803). TXA was not associated with functional outcome (OR 0.96, 95% CI: 0.72–1.27). No potential confounding factors were identified. The proportion good functional outcome in each time category was 226 (56%), 143 (61%) and 101 (61%), for 0–3, 3–6, and >6 h, respectively. See Supplemental Table S4 for good functional outcome by baseline variables. There was no evidence that the effect of TXA on the functional outcome differed by time to treatment (likelihood ratio test: *p* = 0.53) (see Table 4).

**Table 4.** Regression model with effect on good outcome (modified Rankin Scale 0–3) in 803 individuals.

	Number of mRS 0–3 (% of total mRS 0–3)	Odds ratio (95% CI)	*p*-Value,^*^
Tranexamic acid	226 (48.1)	0.96 (0.72–1.27)	0.78
Time category (h)			
0–3	234 (49.8)	1.07 (0.72–1.59)	0.72
3–6	140 (29.8)	1.55 (0.75–3.19)	0.24
>6	90 (19.2)	1.41 (0.67–2.98)	0.37

CI; confidence interval.

Time category: missing in 10 (1.2%).

* Wald-test.

After excluding rebleeding occurring before diagnosis, in an analysis on 768 patients, the impact of TXA on functional outcome was OR 1.02, 95% CI: 0.76–1.36) with otherwise no relevant differences compared to the original analysis (see Table S5).

## Discussion

This post hoc analysis of aSAH patients of the ULTRA-trial provides evidence suggesting that the interval between the (estimated) time of the initial hemorrhage and the initiation of TXA treatment impacts the rebleed rate. The data indicate only a protective effect of TXA on rebleeding when started after at least 6 h after the initial hemorrhage. However, due to the low number of rebleeding in this time category, its overall impact on the total rebleed rate is minimal. The interval between the (estimated) time of initial hemorrhage and the start of TXA treatment does not influence the effect of TXA on functional outcome (mRS) at 6 months.

The results of the current study provide evidence against a reduction in rebleeds with TXA in the aSAH population. This may be attributed to low power of the study or, more likely, the inclusion of very early rebleeds in the current study, which is in line with current treatment protocols where the aSAH is diagnosed as soon as possible.^16^ Because of delay in the diagnosis of aSAH and risk factors that may have a stronger effect on rebleeds than TXA can prevent, the very early and large proportion of rebleeds cannot be prevented by TXA treatment, even if started within 3 h.

Further investigation of known risk factors, such as hypertension in the first hours after the hemorrhage, early surgery for cerebrospinal fluid drainage or emergency aneurysm treatment within 6 h, is therefore warranted to achieve a reduction of the very early rebleeds.^3,22,23^ Unfortunately, most of these data were not collected in the ULTRA-trial, so this hypothesis cannot be investigated.

The failure of showing a positive effect of TXA treatment on outcome has been confirmed consistently by several moderate to high quality randomized controlled trials spanning almost 50 years, in several countries and even with optimized treatment protocols.^11^ This supports the reliability and generalizability of the conclusion of the current study to the complete aSAH population.

Although our analysis suggests that TXA reduces rebleeding if administered later than 6 h after the initial hemorrhage, the absolute number of rebleedings in this time category is low. This low number of rebleeds together with the post-hoc analysis and multiple testing could have caused a statistically significant finding which is a results of chance, instead of a real effect. Moreover, with this low number of rebleeds after 6 h, the protective effect of TXA treatment is applied to a proportion too small to have a significant impact on the overall rebleed rate and functional outcome. This potential protective effect after 6 h contrasts with studies on other bleeding conditions, which showed only a protective effect of TXA treatment when given within 3 h.^12,18,19^ One explanation lies in the differences among underlying pathophysiologies. The goal of TXA treatment in aSAH patients is to stabilize the blood clot to prevent a single devastating rebleeding, while in other indications it primarily delays continuous bleeding. It appears that there are risk factors exerting a stronger effect on rebleeds in the first hours in aSAH than TXA can effectively prevent. Consequently, it is crucial to investigate other risk factors for rebleeds, and treatment protocols should be adjusted, particularly to reduce the rebleeds within the first 6 h after the initial hemorrhage.

This study’s strengths lie in its trial design, with randomization between groups, strict outcome definitions assessed with minimal bias, and the highly precise estimation of the times of randomization and the occurrence of the rebleeding.

The equal distribution of all baseline and time interval variables across groups indicate a low risk for confounding, which may also be extrapolated to unmeasured variables. The low amount of missing data, evenly distributed among groups, mitigated the risk of selection bias. However, a notably high proportion of deaths among the missing data on aneurysm location was observed. This can be attributed to the severity of the initial hemorrhage leading to reduced brain perfusion and making it difficult to ascertain the exact location of the aneurysm.

A notable weakness of this study is its post hoc analysis, resulting in low statistical power. The effect estimates are imprecise with wide confidence intervals. In a strict sense, the results serve as a basis for generating hypotheses and should be interpreted with care regarding the implementation in daily practice.^24,25^ The calculation of estimated times, applicable in 10% of the instances of time of initial hemorrhage, could have introduced information bias. We used strict definitions for calculating the time of the initial hemorrhage, with a maximum imprecision of 3 h in either direction. Assuming to result in non-differential misclassification, this issue likely had a minor impact on the overall results, except for a dilution of the treatment effect. Additionally, the creation of the three time interval groups may have introduced some misclassification. Given the anticipated higher information bias associated with continuous parameters, the decision to analyze in time groups is considered a suitable solution.

Tranexamic acid is an agent extensively studied in various medical contexts, including aSAH, surgery, trauma and bleeding disorders. While numerous studies have demonstrated its benefits, others have shown no efficacy or even potential harm.^19,26^ A more comprehensive understanding of the exact hemostatic and fibrinolytic pathways in various situations could shed light on the observed variations between groups. Increased knowledge of these pathways may facilitate a more detailed exploration of risk factors for rebleeds, potentially leading to the development of novel treatment methods.

It remains plausible that the start of TXA treatment in the ULTRA-trial may still have not been early enough. An alternative approach could be administration of TXA in the ambulance following confirmation of aSAH. Confirming the diagnosis of aSAH is of utmost importance given the potential harm of antifibrinolytics in ischemic stroke, which may present similarly as aSAH. Cranial CT investigations that can be performed in the ambulance or other innovative techniques for investigating intracerebral hemorrhage are therefore necessary.

In conclusion, this study suggests a different effect of TXA on the reduction of rebleeding across different time intervals between the (estimated) time of initial hemorrhage and the start of TXA treatment. Although TXA may demonstrate only a protective effect on rebleeding when started later than 6 h after the initial hemorrhage, its overall influence on the total rebleed rate is minimal with no effect on functional outcome. Other risk factors for very early rebleeds, and the options to reduce these, need to be investigated.

## Supplemental Material

sj-docx-1-eso-10.1177_23969873241246591 – Supplemental material for Impact of time to start of tranexamic acid treatment on rebleed risk and outcome in aneurysmal subarachnoid hemorrhage Supplemental material, sj-docx-1-eso-10.1177_23969873241246591 for Impact of time to start of tranexamic acid treatment on rebleed risk and outcome in aneurysmal subarachnoid hemorrhage by Menno R Germans, Maud A Tjerkstra, René Post, Amy Brenner, Mervyn DI Vergouwen, Gabriël JE Rinkel, Yvo BWEM Roos, René van den Berg, Bert A Coert, W Peter Vandertop and Dagmar Verbaan in European Stroke Journal
