# Supplementary material for: Impact of time to start of tranexamic acid treatment on rebleed risk and outcome in aneurysmal subarachnoid hemorrhage
Source: Eur Stroke J. 2024 Apr 12;9(3):658–66. doi: 10.1177/23969873241246591 (PMC11418415; doi:10.1177/23969873241246591)
Supplement: sj-docx-1-eso-10.1177_23969873241246591 – Supplemental material for Impact of time to start of tranexamic acid treatment on rebleed risk and outcome in aneurysmal subarachnoid hemorrhage [file sj-docx-1-eso-10.1177_23969873241246591.docx]

**SUPPLEMENTAL MATERIAL**

**Assessment of times**

1. The exact time of initial hemorrhage. If the time of hemorrhage was registered as uncertain (e.g. “around 3 p.m.”), the reported time was used with a comment that it was estimated. However, this time is sometimes not known, because the individual had a loss-of-consciousness or a disturbed short-term memory due to the acute hemorrhage. In these cases the time of initial hemorrhage was approximated by the following rules:

- if there was no time retrievable, but it was registered as a part of the day (e.g. morning, afternoon, evening, night), the time was approximated as followed:
  - parts of the day were defined as:
    - morning: 6 a.m. – 12 p.m., with average 9 a.m.
    - afternoon: 12 p.m. – 6 p.m., with average 3 p.m.
    - evening: 6 p.m. – 12 a.m., with average 9 p.m.
    - night: 12 a.m. – 6 a.m., with average 3 a.m.
  - the midpoint between time of admission to first hospital and earliest time of the part of day in question was registered (e.g. ‘bled in the afternoon’ and admitted at 2 p.m. is being registered as midpoint between 2 p.m. and 12 p.m.= 1 p.m.)
  - if the reported part of the day is not the same part of the day of the time of first admission, then the average time of the part of the day in question was registered (e.g. patient bled ‘in the morning’ and admitted at 2 p.m. is being registered as 9 a.m.)
- if there is no information about a time of hemorrhage, the midpoint between ‘last seen well’ and ‘discovered’ was used, as long as it was within 24 hours before admission (according to inclusion criteria)
- if ‘last seen well’ or ‘discovered’ was missing, but individual was transported by ambulance after an emergency phone call, the presentation at first hospital minus 30 minutes was registered as approximated time;

1. The time of admission to first hospital was retrieved from hospital/emergency department or ambulance charts;
2. The time of diagnosis was retrieved from the data on the CT, which routinely registers the time of the investigation;
3. The time of randomization as registered by the randomization software;
4. The time of rebleed (if available) was registered as exact time, or estimated by the first CT following the rebleed;
   - if a high suspicion of a rebleed was present before the diagnosis, the time was estimated from the comments provided by the local investigators or estimated by the admission CT minus 30 minutes
5. The time of aneurysm treatment was defined as the start of anaesthesia for the treatment of the aneurysm.

**Figure S1: Trial allocation profile (CONSORT)**

**Randomized (n=955)**

Excluded (n=142)

- Non-aneurysmal SAH (n=135)
- No (adequate) angiographic imaging obtained (n=7)

**aSAH randomized (n=813)**

## Allocation

**Allocated to tranexamic acid group (n=409)**

- Received TXA treatment (n=395)
- Unknown if TXA was given (n=1)
- Did not receive TXA treatment (n=16)

Immediate aneurysm treatment (n=5)

Physician refused due to medical history (n=1)

Exclusion criteria met after randomization (n=1)

Patient refused TXA treatment (n=1)

Unknown reason (n=8)

**Allocated to control group (n=404)**

- Received no TXA treatment (n= 418)
- Did not receive allocated intervention (n=2)

Demanded tranexamic acid treatment (n=1)

TXA started in ambulance (n=1)

## As-treated

## rebleed analysis

**Analyzed (n=394)**

**Analyzed (n=418)**

## Six months

## follow-up

**Lost to follow-up (n=5)**

- Withdrawal of consent (n=4)
- Patient of caregiver not reachable (n=1)

**Lost to follow-up (n=4)**

- Withdrawal of consent (n=2)
- Caregiver refused (n=1)
- Miscoded as deceased (n=1)

## As-treated

## outcome analysis

**Table S1:** Time intervals in each time category

**Analyzed (n=390)**

**Analyzed (n=413)**

| **Time category (hours)** | **TXA and usual care**  **(n= 394, 48.5%)** | **Usual care only**  **(n= 418, 51.5%)** |
| --- | --- | --- |
| **0 to 3** | 129 (100-151) | 131 (107-152) |
| **3 to 6** | 256 (214-299) | 258 (214-293) |
| **> 6** | 586 (419-982) | 608 (454-959) |
|  |  |  |

Time interval between (estimated) time of initial hemorrhage and (surrogate for) start of TXA treatment. See text for more details.

Data presented as medians (interquartile ranges) and in minutes.

TXA tranexamic acid

**Table S2.** Rates and rate ratios of rebleeds in 811 patients with aneurysmal subarachnoid hemorrhage

| **Variable** | **Category** | **Rebleed** | **Rebleed rate**  **(95% CI)** | **Rate ratio**  **(95% CI)** | **p-value^*^** |
| --- | --- | --- | --- | --- | --- |
|  |  | N=146 | **per person-day** |  |  |
| **Randomization group** | Usual care only  TXA + usual care | 82 (56.2)  64 (43.8) | 0.11 (0.09-0.13)  0.08 (0.07-0.11) | Ref  0.80 (0.58-1.11) | 0.18 |
| **Age** | 20-49 years  50-69 years  ≥70 years | 37 (25.3)  84 (57.5)  25 (17.1) | 0.11 (0.08-0.16)  0.13 (0.11-0.17)  0.04 (0.03-0.06) | Ref  1.18 (0.81-1.74)  0.38 (0.23-0.63) | <0.001 |
| **Sex** | Male  Female | 46 (31.5)  100 (68.5) | 0.10 (0.07-0.13)  0.09 (0.08-0.11) | Ref  0.96 (0.68-1.37) | 0.83 |
| **WFNS**^†^ | I  II  III  IV  V  I-III  IV-V | 32 (21.9)  29 (19.9)  11 (7.5)  35 (24.0)  36 (24.7)  72 (49.3)  71 (50.7) | 0.05 (0.03-0.07)  0.10 (0.07-0.15)  0.27 (0.15-0.49)  0.11 (0.08-0.15)  0.17 (0.13-0.24)  0.07 (0.06-0.09)  0.13 (0.11-0.17) | Ref  2.13 (1.29-3.52)  5.63 (2.84-11.17)  2.23 (1.38-3.61)  3.63 (2.25-5.84)  Ref  1.83 (1.30-2.55) | <0.001  <0.001 |
| **Fisher grade** | II/III^\|\|^  IV | 26 (17.8)  120 (82.2) | 0.05 (0.03-0.07)  0.12 (0.10-0.15) | Ref  2.71 (1.71-4.14) | <0.001 |
| **Medication use** | Platelet inhibitor  Anticoagulation | 19 (13.0)  3 (2.1) | 0.09 (0.06-0.14)  0.01 (0.00-0.04) | 0.92 (0.57-1.49)^‡^  0.13 (0.04-0.40)^§^ | 0.73  <0.001 |
| **Location of aneurysm**^#^ | Anterior  Posterior | 97 (68.8)  44 (31.2) | 0.12 (0.09-0.14)  0.07 (0.05-0.09) | Ref  0.61 (0.43-0.87) | 0.01 |
| **Treatment modality** | Endovascular  Clipping  None | 73 (50.0)  25 (17.1)  48 (32.9) | 0.12 (0.10-0.16)  0.10 (0.06-0.14)  0.07 (0.05-0.10) | Ref  0.77 (0.49-1.21)  0.58 (0.40-0.84) | 0.36 |
| **Time category**^**^ | 0-3  3-6  >6 | 87 (59.6)  38 (26.0)  21 (14.4) | 0.13 (0.11-0.16)  0.09 (0.06-0.12)  0.05 (0.03-0.08) | Ref  0.66 (0.45-0.96)  0.38 (0.23-0.61) | <0.001 |
| **Treatment center** | A  B  C  D  E  F  G  H  other | 73 (50.0)  30 (20.5)  14 (9.6)  12 (8.2)  10 (6.8)  2 (1.4)  4 (2.7)  0  1 (0.7) | 0.11 (0.09-0.13)  0.10 (0.07-0.14)  0.06 (0.03-0.10)  0.17 (0.10-0.31)  0.09 (0.07-0.16)  0.06 (0.02-0.25)  0.08 (0.03-0.22)  -  0.60 (0.08-4.23) | Ref  0.93 (0.61-1.42)  0.53 (0.30-0.94)  1.63 (0.88-3.00)  0.81 (0.42-1.56)  0.59 (0.15-2.42)  0.78 (0.29-2.14)  -  5.57 (0.77-40.08) | 0.35 |
|  |  |  |  |  |  |

Ref Reference, TXA tranexamic acid

^*^ χ^2^-test

^†^ WFNS score: missing in 10 (1.2%)

^‡^ reference category is no platelet inhibitors

^§^ reference category is no anticoagulation

^||^ Fisher II and III were combined, because only one rebleed occurred in Fisher II category

^#^ Aneurysm location: missing in 5 (0.6%)

^**^ Time category: missing in 8 (1.0%)

**Table S3.** Cox regression model with effect on rebleeding after excluding rebleeds before diagnosis (sensitivity analysis)

| **Variable** | **Number of rebleeds**  **(% of total rebleeds)** | **Hazard ratio**  **(95% CI)** | **p-value^*^** |
| --- | --- | --- | --- |
| **Tranexamic acid** | 46 (41.8) | 0.80 (0.55-1.16) | 0.24 |
| **Effect of TXA per**  **time category**  0-3  3-6  > 6 | 79 (55.2)  38 (26.6)  18 (12.6) | 0.85 (0.53-1.36)  0.53 (0.23-1.25)  0.19 (0.06-0.55) | 0.50  0.15  0.02 |
|  |  |  |  |

CI confidence interval, TXA tranexamic acid

^*^ Wald-test

**Table S4.** Functional outcome of 803 patients with aneurysmal subarachnoid hemorrhage

| **Variable** | **Category** | **Functional outcome** | | **Odds ratio^*^**  **(95% CI)** | **p-value**^†^ |
| --- | --- | --- | --- | --- | --- |
|  |  | **mRS 0-3** | **mRS 4-6** |  |  |
|  |  | N = 470 | N = 333 |  |  |
| **Randomization group** | Usual care only  TXA + usual care | 244 (50.5)  226 (49.5) | 169 (50.8)  164 (49.2) | Ref  0.96 (0.72-1.27) | 0.78 |
| **Age** | 20-49 years  50-69 years  ≥70 years | 147 (31.3)  276 (58.7)  47 (10.0) | 64 (19.2)  183 (55.0)  86 (25.8) | Ref  0.66 (0.46-0.93)  0.24 (0.15-0.39) | <0.001 |
| **Sex** | Male  Female | 130 (27.7)  340 (72.3) | 102 (30.6)  231 (69.4) | Ref  1.15 (0.85-1.57) | 0.39 |
| **WFNS** | I  II  III  IV  V  I-III  IV-V | 224 (48.1)  100 (21.5)  18 (3.9)  86 (18.5)  38 (8.2)  343 (73.5)  124 (26.6) | 46 (14.1)  51 (15.6)  20 (6.1)  92 (28.1)  118 (36.1)  117 (3.8)  210 (64.2) | Ref  0.40 (0.25-0.65)  0.18 (0.09-0.39)  0.19 (0.12-0.31)  0.07 (0.04-0.12)  Ref  0.20 (0.15-0.28) | <0.001  <0.001 |
| **Fisher grade** | II/III  IV | 205 (43.6)  265 (56.4) | 50 (15.0)  283 (85.0) | Ref  0.23 (0.16-0.33) | <0.001 |
| **Medication use** | Platelet inhibitor  Anticoagulation | 43 (9.2)  5 (1.1) | 55 (16.5)  22 (6.6) | 0.50 (0.33-0.77)^‡^  0.15 (0.06-0.41)^§^ | 0.001  0.04 |
| **Location of**  **aneurysm** | Anterior  Posterior | 321 (68.9)  145 (31.1) | 208 (65.0)  112 (35.0) | Ref  0.84 (0.62-1.14) | 0.28 |
| **Treatment modality** | Endovascular  Clipping  None | 354 (75.5)  105 (22.4)  10 (2.1) | 168 (50.5)  68 (20.4)  97 (29.1) | Ref  0.73 (0.51-1.05)  0.05 (0.02-0.10) | <0.001 |
| **Time category** | 0-3 hrs.  3-6 hrs.  >6 hrs. | 226 (48.1)  143 (30.4)  101 (21.5) | 177 (53.2)  92 (27.2)  64 (19.2) | Ref  1.22 (0.88-1.69)  1.24 (0.85-1.79) | 0.37 |
| **Treatment center** | A  B  C  D  E  F  G  H  other | 202 (43.0)  92 (19.6)  72 (15.3)  30 (6.4)  29 (6.2)  29 (6.2)  14 (3.0)  2 (0.4)  0 | 154 (46.3)  66 (19.8)  56 (16.8)  22 (6.6)  15 (4.5)  5 (1.5)  10 (3.0)  3 (0.9)  2 (0.6) | Ref  1.06 (0.73-1.55)  0.98 (0.65-1.47)  1.04 (0.58-1.87)  1.47 (0.76-2.85)  4.42 (1.65-11.86)  1.07 (0.46-2.47)  0.51 (0.08-3.09)  n/a | 0.97 |
|  |  |  |  |  |  |

Data presented as n (%). Percentages may not total 100 because of rounding. mRS Modified Rankin Scale, n/a not applicable, TXA tranexamic acid, WFNS World Federation of Neurosurgical Societies

^*^ Odds ratio for good outcome (mRS 0-3)

^†^ χ^2^-test

^‡^ reference category is no platelet inhibitors

^§^ reference category is no anticoagulation

Missings: WFNS: 10 (1.2%); Aneurysm location: 17 (2.1%); Treatment modality: 1 (0.1%)

**Table S5.** Final model with effect on functional outcome (modified Rankin Scale 0-3) after excluding rebleeds before diagnosis (sensitivity analysis)

| **Variable** | **Number of mRS 0-3**  **(% of total mRS 0-3)** | **Hazard ratio**  **(95% CI)** | **p-value^*^** |
| --- | --- | --- | --- |
| **Tranexamic acid** | 210 (47.5) | 1.02 (0.76-1.36) | 0.92 |
| **Effect of TXA per**  **time category**  0-3 hrs.  3-6 hrs.  > 6 hrs. | 100 (47.6)  68 (32.4)  42 (20.0) | 1.12 (0.74-1.70)  1.63 (0.76-3.46)  1.51 (0.69-3.28) | 0.58  0.20  0.30 |
|  |  |  |  |

CI confidence interval

^*^ Wald-test

ULTRA coinvestigators

| First name | Initial(s) | Surname | Degree | Role | Contribution |
| --- | --- | --- | --- | --- | --- |
| Frank |  | de Beer^1^ | MD | SI | data collection, manuscript review |
| Frits | C | de Beer^2^ | MD | SI | data collection, manuscript review |
| Renske | M | van den Berg-Vos^3^ | PhD | SI | data collection, manuscript review |
| Henri | P | Bienfait^4^ | PhD | SI | data collection, manuscript review |
| Hieronymus | D | Boogaarts^5^ | PhD | SI | data collection, manuscript review |
| Irene |  | Bronner^6^ | PhD | SI | data collection, manuscript review |
| Paul | J A M | Brouwers^7^ | PhD | SI | data collection, manuscript review |
| Janneke |  | Horn^8^ | PhD | LI | data collection, manuscript review |
| Korné |  | Jellema^9^ | PhD | SI | data collection, manuscript review |
| Hans |  | Kieft^10^ | PhD | SI | data collection, manuscript review |
| Catharina | J M | Klijn^11^ | PhD | SI | data collection, manuscript review |
| Radboud | W | Koot^12^ | PhD | SI | data collection, manuscript review |
| Nyika | D | Kruyt^13^ | PhD | SI | data collection, manuscript review |
| Vincent | I H | Kwa^3^ | PhD | LI | data collection, manuscript review |
| Charles | B L M | Majoie^14^ | PhD | SI | data collection, manuscript review |
| Dharmin |  | Nanda^2^ | PhD | SI | data collection, manuscript review |
| Bram |  | van der Pol^15^ | PhD | SI | data collection, manuscript review |
| Taco | C | van der Ree^16^ | PhD | SI | data collection, manuscript review |
| Loes | J A | Reichman^17^ | MD | SI | data collection, manuscript review |
| Gerwin |  | Roks^18^ | PhD | SI | data collection, manuscript review |
| Janneke |  | van den Vlekkert^6^ | PhD | SI | data collection, manuscript review |
| Jasper | F C | Wolfs^19^ | PhD | SI | data collection, manuscript review |

LI local investigator, SI site investigator

1 Department of Neurology, Spaarne Gasthuis, Haarlem, the Netherlands

2 Department of Neurosurgery, Isala Hospital, Zwolle, the Netherlands

3 Department of Neurology, Onze Lieve Vrouwe Gasthuis, Amsterdam, the Netherlands

4 Department of Neurology, Gelre Hospital, Apeldoorn, The Netherlands

5 Department of Neurosurgery, Radboud University Medical Center, Nijmegen, the Netherlands

6 Department of Neurology, Flevo Hospital, Almere, the Netherlands

7 Department of Neurology, Medisch Spectrum Twente, Enschede, the Netherlands

8 Department of Intensive Care, Amsterdam University Medical Centers, location AMC, Amsterdam, the Netherlands

9 Department of Neurology, Haaglanden Medical Center, The Hague, the Netherlands

10 Department of Intensive Care, Isala Hospital, Zwolle, the Netherlands

11 Department of Neurology, Donders Institute for Brain, Cognition and Behaviour, Radboud University Medical Center, Nijmegen, the Netherlands

12 Department of Neurosurgery, Leids University Medical Center, the Netherlands

13 Department of Neurology, Leids University Medical Center, the Netherlands

14 Department of Radiology and Nuclear Medicine, Amsterdam University Medical Centers, location AMC, Amsterdam, the Netherlands

15 Department of Neurosurgery, Elisabeth Tweesteden ziekenhuis, Tilburg, the Netherlands

16 Department of Neurology, Dijklander Hospital, Hoorn, the Netherlands

17 Department of Neurology, Ziekenhuisgroep Twente, Almelo, the Netherlands

18 Department of Neurology, Elisabeth Tweesteden ziekenhuis, Tilburg, the Netherlands

19 Department of Neurosurgery, Haaglanden Medical Center, The Hague, the Netherlands
